# Supplementary material for: Development of human cGAS-specific small-molecule inhibitors for repression of dsDNA-triggered interferon expression
Source: Nat Commun. 2019 May 21;10:2261. doi: 10.1038/s41467-019-08620-4 (PMC6529454; doi:10.1038/s41467-019-08620-4)
Supplement: Supplementary file 1 — Supplementary Information [file 41467_2019_8620_MOESM1_ESM.pdf]

Supplementary Information

Development of human cGAS-specific small-molecule inhibitors for repression of dsDNA-triggered interferon expression

Lama et al.

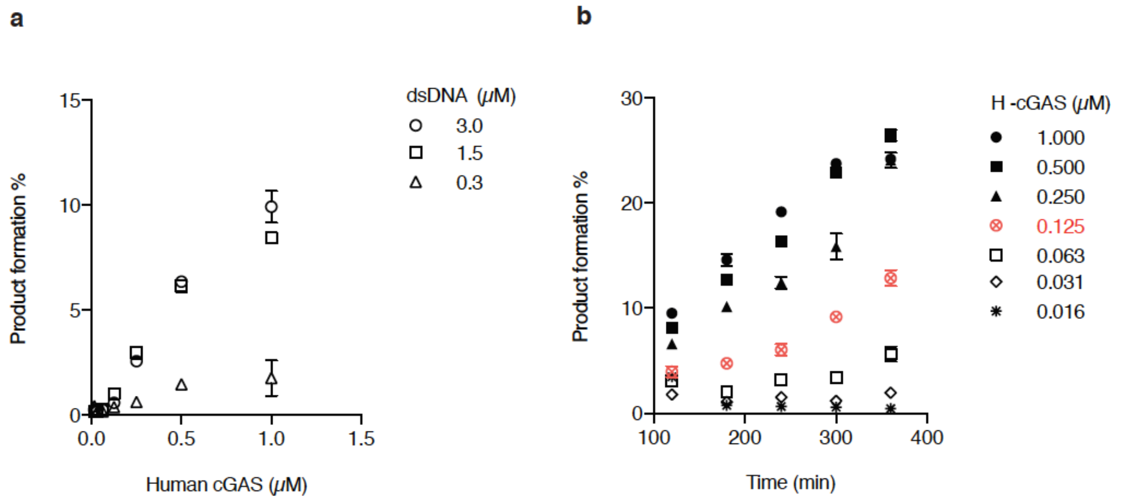

Supplementary Figure 1 Development and optimization of luminescence based high throughput screening assay for human cGAS (**a**) Titration of h-cGAS at 3 different concentrations of 45-bp dsDNA. The reactions were performed for 120 min. (**b**) Time course analysis of h-cGAS activity using 50 nM 100-bp dsDNA, 300 μM ATP, and 300 μM GTP. The activity of cGAS enzyme was determined by calculating % cGAMP product formation using an RF-MS assay based on consumption of ATP and GTP, and the generation of cGAMP. (n = 3; mean ± S.D.)

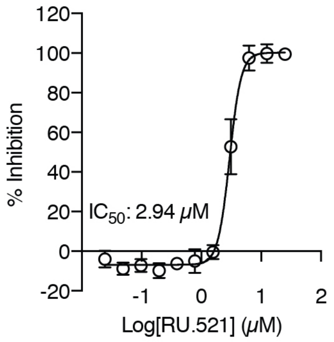

Supplementary Figure 2 in vitro concentration response curve for RU.521 using recombinant h-cGAS. IC_{50} value was determined for the inhibitor using RF-MS based assay. (*n* = 3; mean ± S.D.; Data shown are representation of two independent experiments.)

a

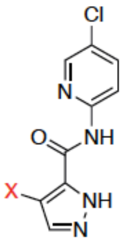

Molecule ID	X	h-cGAS IC_{50} (μM)	m-cGAS IC_{50} (μM)
J001	NO_2	1.03	0.30
J063	CF_3	>25.0	>25.0
J064	CN	>25.0	>25.0
J065	COOH	>25.0	>25.0
J067	$CONH_2$	>25.0	>25.0
J006	Cl	>25.0	>25.0
J009	CHO	>25.0	>25.0
J008	H	>25.0	>25.0
J007	Me	>25.0	>25.0
J069	NH_2	>25.0	21.9

b

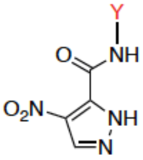

Molecule ID	Y	h-cGAS IC_{50} (μM)	m-cGAS IC_{50} (μM)
J001	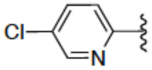	1.03	0.30
J005	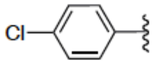	>25.0	18.6
J038	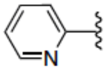	1.86	0.58
J046	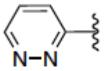	4.62	2.41
J043	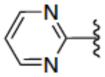	2.39	1.89
J037	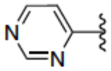	1.75	0.98
J036	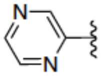	1.42	0.43

Supplementary Figure 3 (**a**) Evaluation of nitro group on pyrazole ring in **J001** through replacement with other functional groups. (**b**) Evaluation of pyridine ring in **J001** through replacement with other 6-membered rings.

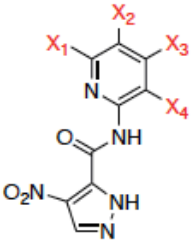

Molecule ID	X_1	X_2	X_3	X_4	h-cGAS IC_{50} (μM)	m-cGAS IC_{50} (μM)
J106	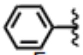	H	H	H	>25.0	>25.0
J050	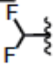	H	H	H	10.2	2.70
J047	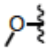	H	H	H	5.00	1.70
J049	Cl–	H	H	H	6.28	1.33
J044	H_3C–	H	H	H	2.76	0.65
J015	H	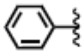	H	H	0.20	0.10
J025	H	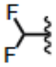	H	H	0.65	0.39
J024	H	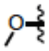	H	H	0.59	0.41
J001	H	Cl–	H	H	1.04	0.49
J032	H	H_3C–	H	H	0.84	0.26
J014	H	H	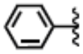	H	0.10	0.06
J019	H	H	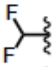	H	0.23	0.06
J021	H	H	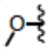	H	0.31	0.08
J022	H	H	Cl–	H	0.38	0.13
J031	H	H	H_3C–	H	1.11	0.28
J057	H	H	H	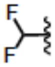	>25.0	14.9
J054	H	H	H	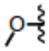	>25.0	16.3
J056	H	H	H	Cl–	>25.0	10.3
J053	H	H	H	H_3C–	>25.0	>25.0

Supplementary Figure 4 Evaluation of substituents on pyridine ring in **J001** through addition of different substituents on four available carbon positions on pyridine ring.

a

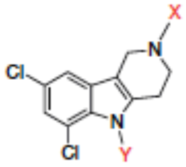

Molecule ID	X	Y	h-cGAS IC_{50} (μM)	m-cGAS IC_{50} (μM)
G001	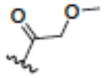	H	2.08	0.44
G002	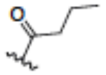	H	>25.0	>25.0
G004	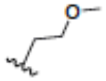	H	>25.0	>25.0
G007	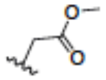	H	>25.0	>25.0
G008	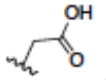	H	>25.0	>25.0
G010	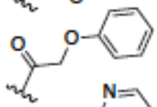	H	>25.0	>25.0
G014	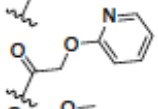	H	>25.0	>25.0
G003	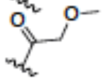	Me	2.41	0.774
G009	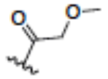	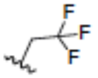	13.9	3.45

b

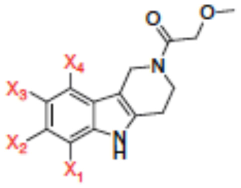

Molecule ID	X_1	X_2	X_3	X_4	h-cGAS IC_{50} (μM)	m-cGAS IC_{50} (μM)
G015	Cl	Cl	H	H	0.35	0.026
G001	Cl	H	Cl	H	2.08	0.44
G013	Cl	H	H	Cl	3.23	0.67
G025	Br	Cl	H	H	0.203	0.024
G028	Cl	Br	H	H	0.198	0.032
G076	Cl	CN	H	H	13.5	0.92
G078	CN	Cl	H	H	0.795	0.043
G024	Cl	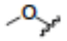	H	H	>25.0	9.78
G026	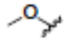	Cl	H	H	24.7	0.70

Supplementary Figure 5 (**a**) Evaluation of methoxy-propanone side chain on piperidine ring or indole amino group in **G001** through replacement with other side chains. (**b**)

Evaluation of chlorine substituents on indole ring in **G001** through replacement with different substituents on the indole ring.

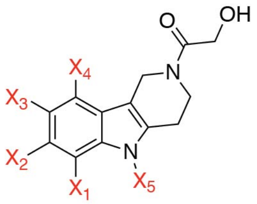

Molecule ID	X_1	X_2	X_3	X_4	X_5	h-cGAS IC_{50} (μM)	m-cGAS IC_{50} (μM)
G022	Cl	Cl	H	H	H	0.106	0.0180
G083	Br	Cl	H	H	H	0.140	0.0280
G085	F	Cl	H	H	H	0.123	0.0210
G090	Me	Cl	H	H	H	0.295	0.0350
G086	Cl	Br	H	H	H	0.0450	0.0150
G089	Cl	F	H	H	H	0.296	0.0750
G088	Cl	Me	H	H	H	0.619	0.0880
G097	F	Cl	H	H	Me	0.138	0.0128
G103	Cl	CN	H	H	H	0.402	0.0450
G092	F	Br	H	H	H	0.0390	0.0180
G105	F	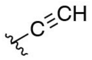	H	H	H	1.45	0.510
G100	F	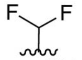	H	H	H	15.8	1.06
G101	F	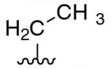	H	H	H	>25.0	21.8
G102	F	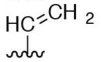	H	H	H	19.0	2.93
G084	Me	Me	H	H	H	6.56	0.445
G104	CN	CN	H	H	H	8.32	0.510
G098	Cl	Cl	H	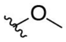	H	0.0490	0.0240
G108	Cl	Cl	H	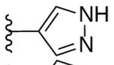	H	0.0275	5.15
G140	Cl	Cl	H	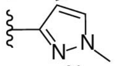	H	0.0140	0.442
G150	Cl	Cl	H	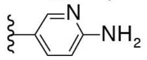	H	0.0102	>25.0

Supplementary Figure 6 Evaluation of different substituents on indole ring in **G022**.

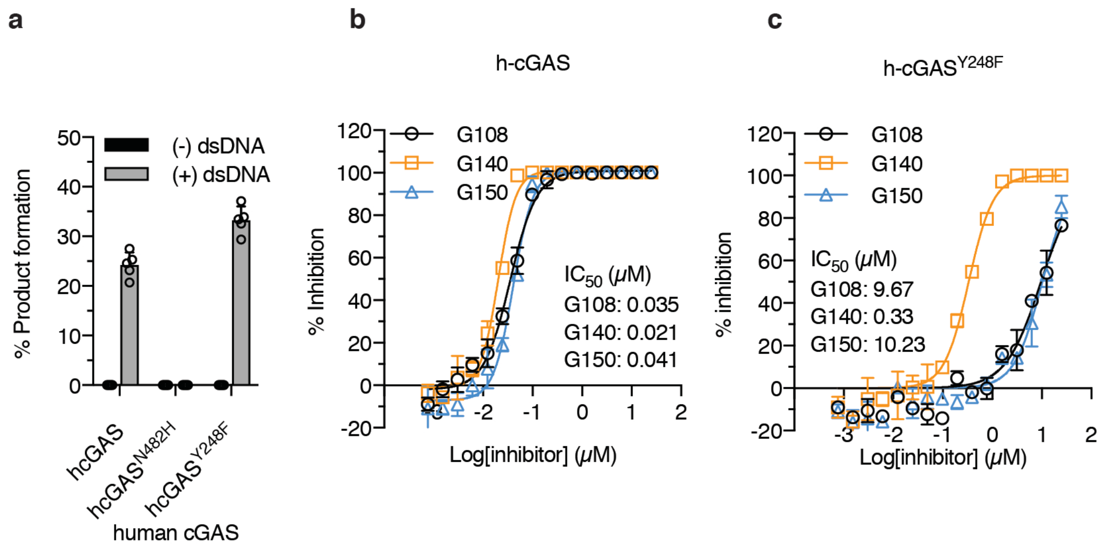

Supplementary Figure 7 Human cGAS (h-cGAS) residue Tyr248 is important for inhibitors specificity. (**a**) Enzymatic activity of native human cGAS, h-$cGAS^{N482H}$, and h-$cGAS^{Y248F}$. (**b, c**) In vitro concentration response curves for **G108**, **G140**, and **G150** using native h-cGAS (**b**) or h-$cGAS^{Y248F}$ (**c**). Enzymatic activity and IC_{50} values were determined using RF-MS based assay. (*n* = 5 for **a**, 3 for **b** and 2 for **c**; mean ± S.D. Data shown are representation of two independent experiments.)

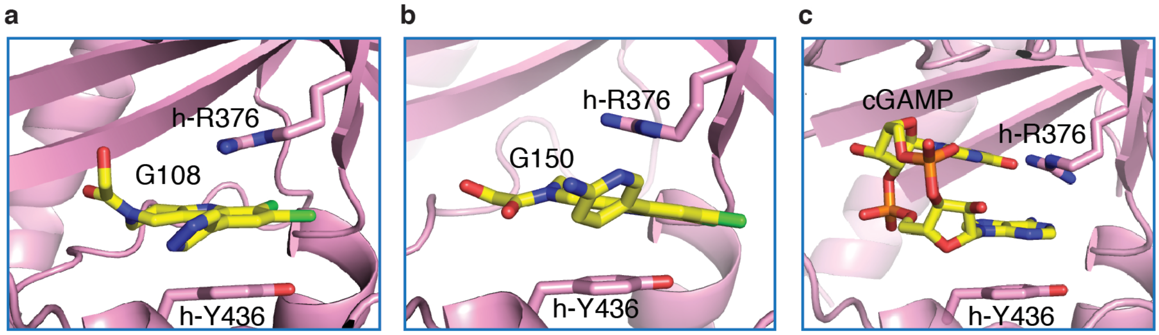

Supplementary Figure 8 Comparison of the alignments of bound (**a**) **G108**, (**b**) **G150** and (**c**) cGAMP in the h-cGAS^{CD} binding pocket. The side chains of Arg376 and Tyr436 are also included in the panels.

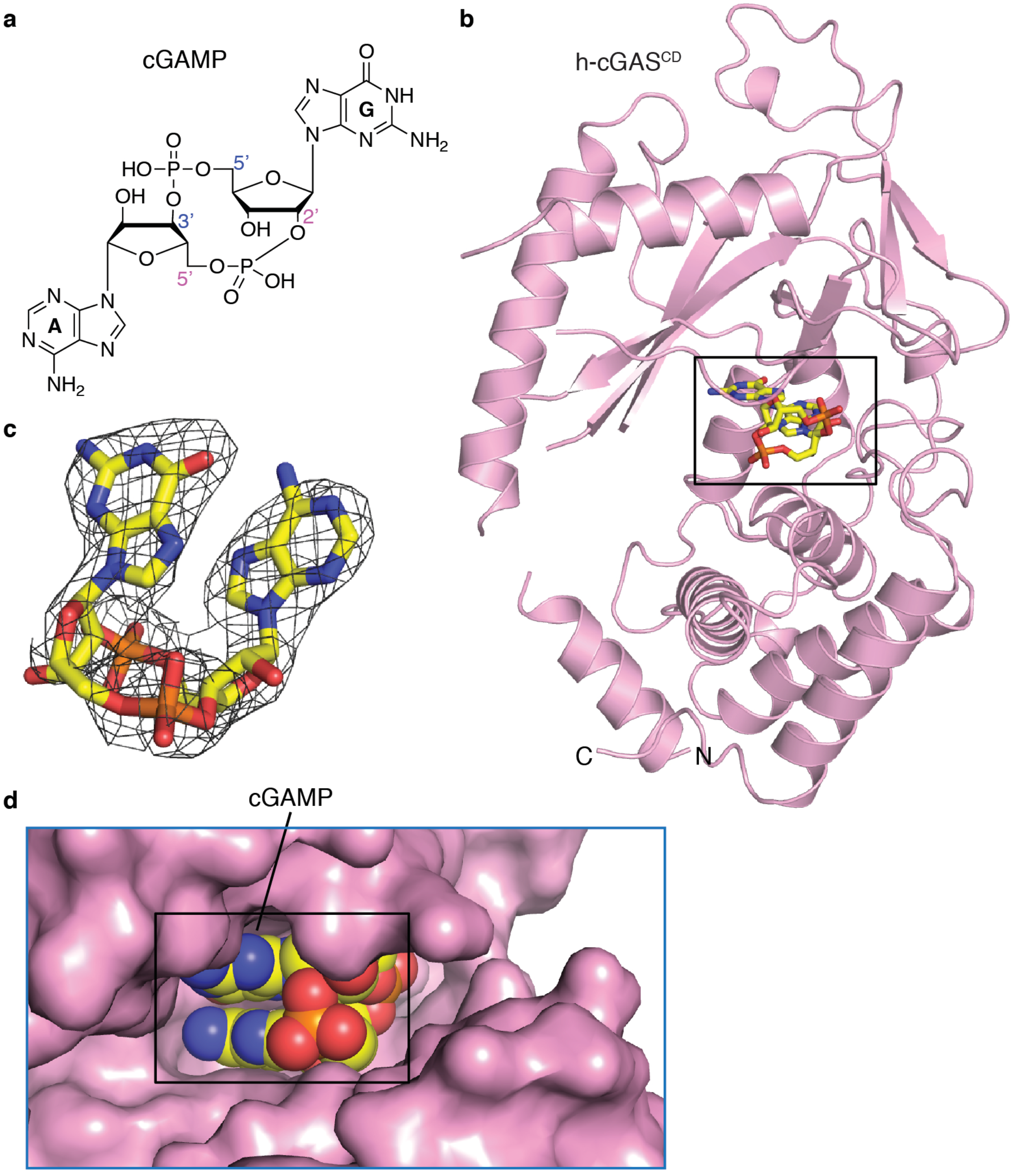

Supplementary Figure 9 Structure of cGAMP bound to apo h-cGAS. (**a**) Chemical formula of cGAMP. (**b**) Crystal structure of cGAMP bound to apo h-cGAS^{CD}. The bound cGAMP is shown in a stick representation and the binding pocket is boxed. (**c**) 2Fo-Fc

electron density map of bound cGAMP contoured at 1.2 σ. (**d**) Positioning of the bound cGAMP towards one end of the extended ligand binding pocket of h-cGAS^{CD}.

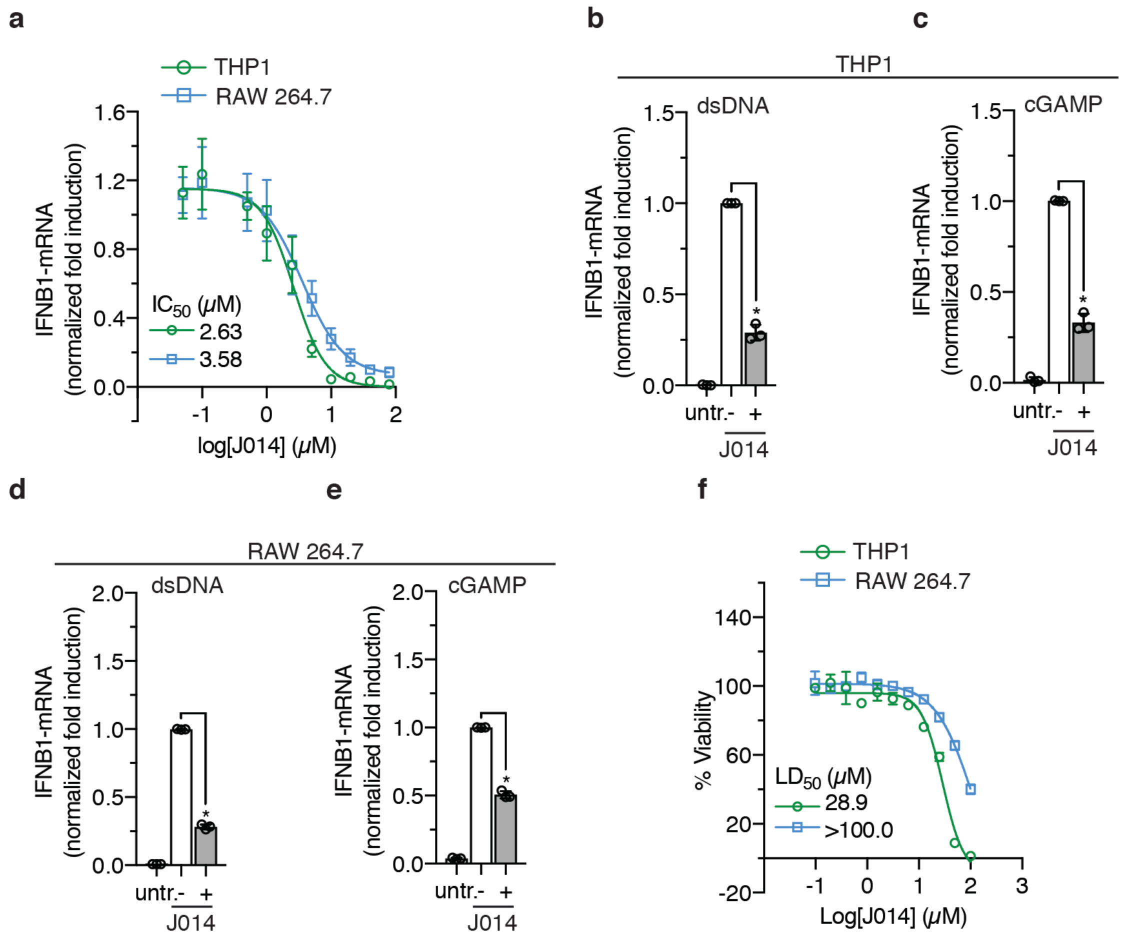

Supplementary Figure 10 Inhibition of cGAS-dependent interferon induction by **J014** in human and murine macrophage cells. (**a**) Cellular inhibitory potency of **J014** against cGAS activity was tested in human THP1 or murine RAW 264.7 cells using dsDNA for cGAS stimulation. *IFNB1* mRNA was measured by qRT-PCR for each of the indicated inhibitor concentrations and normalized to no inhibitor control. (**b, c**) Specificity analysis of 5 µM **J014** against cGAS inhibition in THP1 cells using different ligands: 2 µg ml^{-1} dsDNA (cGAS), and 10 µg ml^{-1} cGAMP (STING). (**d, e**) Specificity analysis of 10 µM **J014** against cGAS inhibition in RAW 264.7 cells using different ligands: 2 µg ml^{-1} dsDNA (cGAS), and 10 µg ml^{-1} cGAMP (STING). Untr., untreated cells. (**f**) Cytotoxic

effect of **J014** was tested in THP1 and RAW 264.7 cells using different concentrations range. (n = 3; mean ± S.D.; *p < 0.001, using two-tailed Student's t-test in **b-e**. Data shown are representation of two independent experiments.)

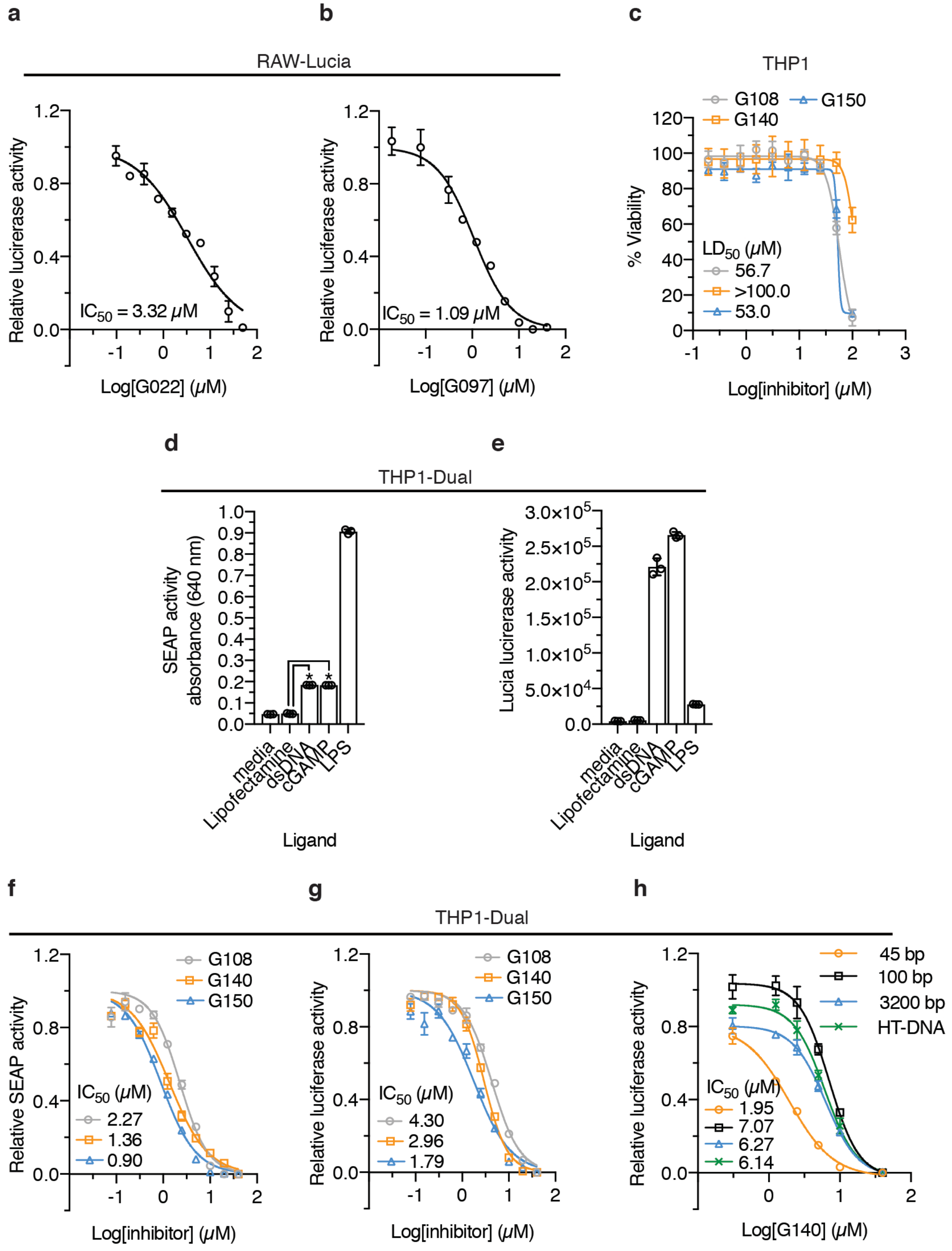

a
b
c
RAW-Lucia
Relative luciferase activity
IC50 = 3.32 μM
Log[G022] (μM)
IC50 = 1.09 μM
Log[G097] (μM)
THP1
G108
G150
G140
% Viability
LD50 (μM)
56.7
>100.0
53.0
Log[inhibitor] (μM)
d
e
THP1-Dual
SEAP activity absorbance (640 nm)
media
Lipofectamine
dsDNA
cGAMP
LPS
Ligand
Lucia luciferase activity
f
g
h
Relative SEAP activity
IC50 (μM)
2.27
1.36
0.90
4.30
2.96
1.79
45 bp
100 bp
3200 bp
HT-DNA
1.95
7.07
6.27
6.14
Log[G140] (μM)

Supplementary Figure 11 Cellular potency analyses of G chemotype cGAS inhibitors in human and murine macrophage cells. (**a, b**) Inhibition of dsDNA-stimulated activity of cGAS by **G022**, and **G097** were tested using a range of inhibitor concentrations in RAW-Lucia cells and measuring the inhibition of Lucia luciferase activity using Quanti-Luc reagent (InvivoGen). (**c**) Cytotoxic effect of **G108**, **G140**, and **G150** were tested in THP1 cells using different concentrations range. (**d, e**) Analysis of activation of NF-κB pathway (**d**) and interferon-inducible gene (**e**) were analyzed in THP1-Dual cells using different ligands: 0.5 μg ml^{-1} dsDNA (cGAS), 2.5 μg ml^{-1} cGAMP (STING), or 25 ng ml^{-1} LPS (TLR4). (**f, g**) Inhibition of dsDNA-stimulated activity of cGAS by **G108**, **G140**, and **G150** were tested using a range of inhibitor concentrations in THP1-Dual cells and measuring the inhibition of SEAP activity using Quanti-Blue reagent (InvivoGen) (**f**) or the inhibition of Lucia luciferase activity using Quanti-Luc reagent (**g**). (**h**) Potency of **G140** was tested in dsDNA-stimulated THP1-Dual cells in the presence of a range of different inhibitor concentrations using 0.5 μg ml^{-1} of dsDNA ligands of different length or herring-testes DNA (HT-DNA). untr., cells treated only with Lipofectamine2000 transfection reagent. Relative luciferease activity or relative SEAP activity were determined with respect to dsDNA stimulated cell samples with no inhibitor control. The cellular IC_{50} values and LD_{50} values were calculated using GraphPad Prism (7.01). (n = 2 for **a** and **b**, 3 for **c-h**; mean ± S.D.; *$p < 0.001$, using one-way ANOVA followed by Tukey's test for multiple comparison (**d**). Data shown are representation of two independent experiments.)

a

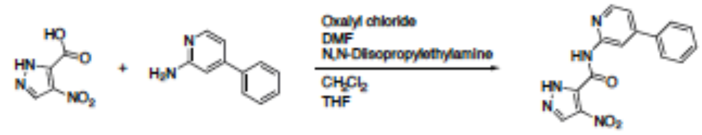

b

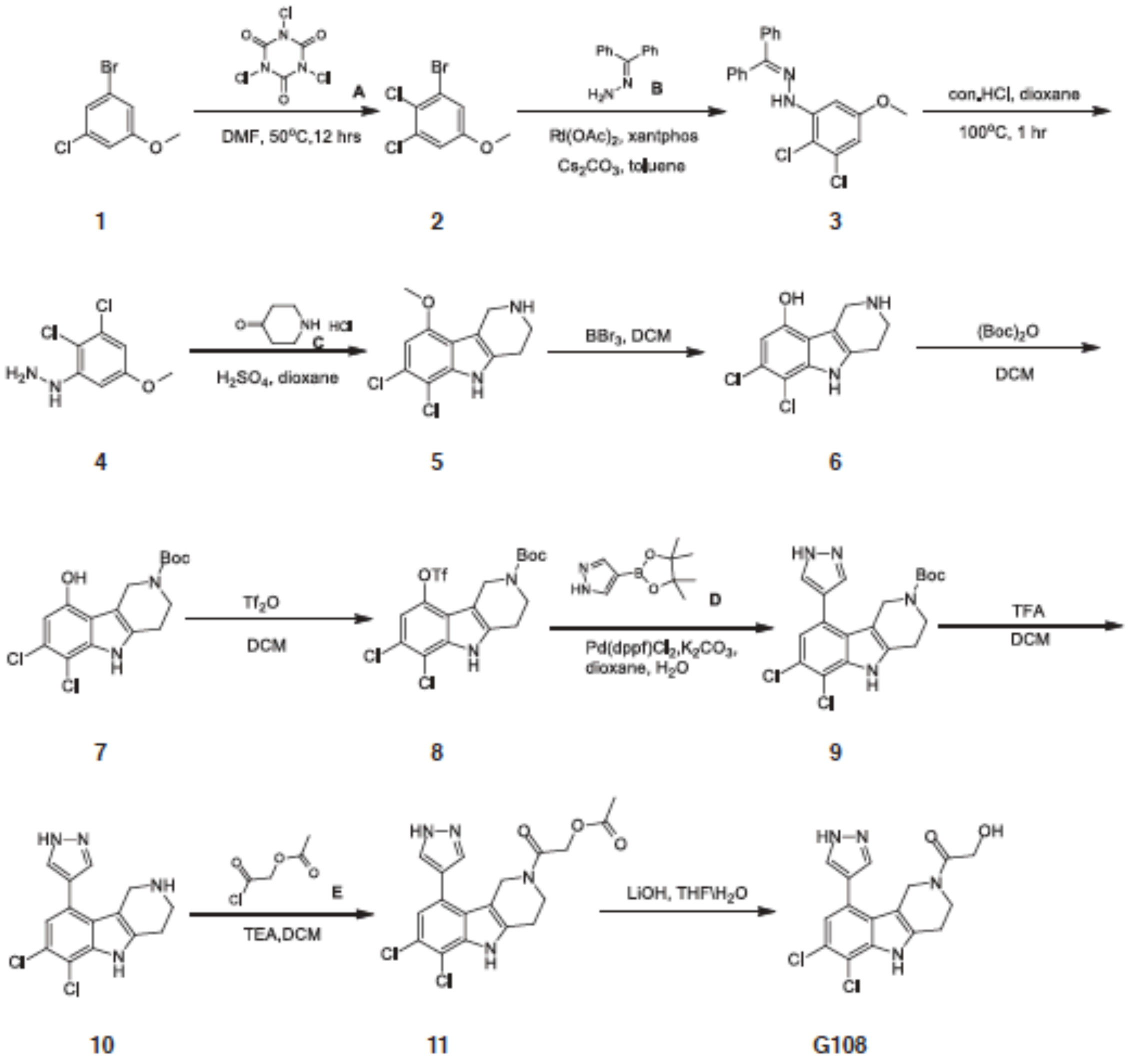

Supplementary Figure 12 Chemical synthesis schemes depicting syntheses of **J014** (**a**) and **G108** (**b**).

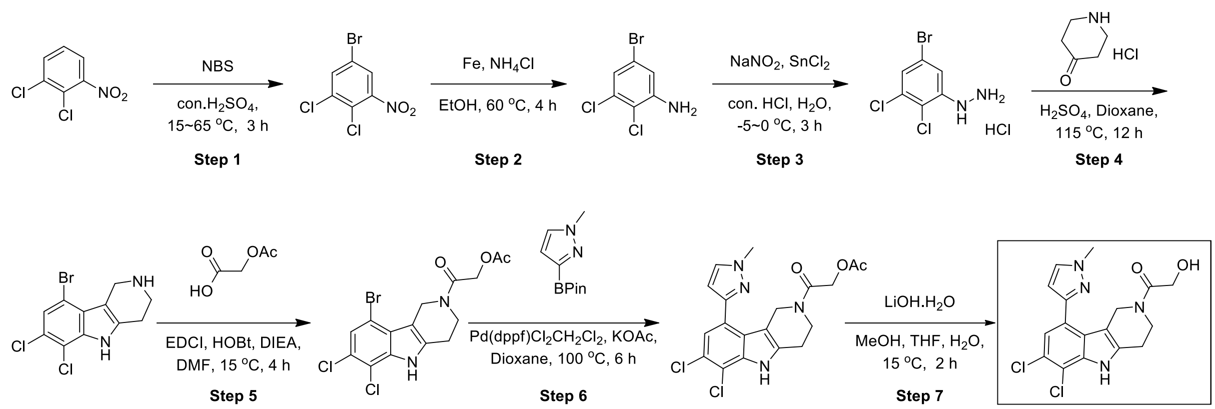

T015T-010-1

Supplementary Figure 13 Chemical synthesis scheme depicting synthesis of **G140** (T015T-010-1).

Category	Parameter	Description
Assay	Type of assay<br>Target<br>Primary measurement<br>Key reagents<br>Assay protocol<br><br><br><br><br><br><br><br><br>Additional comment	Purified enzyme assay<br>Human Cyclic GMP-AMP Synthase (h-cGAS)<br>Luminescence<br>h-cGAS, ATP, GTP, dsDNA<br>20 µL final volume: Buffer of the reaction containing 20 mM Tris-HCl pH 7.4, 150 mM NaCl, 5mM $MgCl_2$, 1mM DTT, 1µM $ZnCl_2$ and 0.01% Tween-20. Samples contains buffer of the reaction, 100 µM ATP and GTP, 25 nM dsDNA and 100 nM h-cGAS incubated for 7 hours at room temperature. The reaction was stopped with 20 µL of Kinase-Glo® Max Luminescent Kinase Assay. Luminescence was measured in a Biotek Synergy Neo plate reader. Order of addition: 10 µL of reaction buffer, 0.05 µL compounds, 5 µL of a mix of ATP, GTP and dsDNA (columns 1-23), 5 µL of a reaction containing ATP, GTP (column 24), 5 µL solution containing h-GAS (full plate). The plates were spun down for 30 seconds at 100xg.
Library	Library size<br>Library composition<br><br>Source<br><br><br><br>Additional comments	281,348 pure compounds<br>Low molecular weight compounds were a random subset from the Rockefeller University Compound Library of 283,000 compounds<br>Analyticon, Biofocus, Cerep, Chembridge, ChemDiv, ChemX-Infinity, NIH Clinical Collection, Edelris, Enamine, Greenpharma, NIH Small Molecule Repository, LifeChem, NIH Clinical Collection, Pharmakon-900, Prestwick, Selleckchem, Specs, Spectrum Chemicals and Tocris.<br>Library description at www.rockefeller.edu/htsrc/
Screen	Format<br>Concentration tested<br>Plate controls<br>Reagent/compound dispensing system<br>Detection instrument and software<br>Assay Validation /QC<br><br><br><br>Correction factors	384-well plate<br>12.5 µM; 0.25% DMSO<br>DMSO (negative control), no dsDNA (positive control)<br>MultiDrop Combi with RapidStack (Thermo Scientific) for reagents; Janus Automated Workstation with Nanohead (Perkin-Elmer) for compounds.<br>Biotek Synergy Neo plate reader, Biotek, Gen5.<br><br>Hit-compounds were re picked from the original 5 mM source and retested at 10 serial diluted concentrations; in triplicate and a concentration

	Normalization data<br><br><br><br><br><br><br>Additional comments	response curved fitted to a 4 parameter hyperbolic using CDD software. HPLC performed to all hits compounds to detect purity.<br>none<br>The % of inhibition is calculated as follow: % inhibition = 100 x [(sample - average negative control)/(average positive control – average negative control)].<br>The quality of the screen was assessed by Z' factor calculated as follows: Z' = 1-[3*(standard deviation positive control + standard deviation negative control)/(average positive control – average negative control)].<br>To determine the percentage of inhibition of h-cGAS the data was normalized against the positive control (column 24) and negative control (column 23).
Post-HTS analysis	Hit criteria<br>Hit rate<br>Additional assays<br>Confirmation of hit purity and structure<br>Additional comments	Normalized percentage of inhibition ≥40%<br>0.08%<br>RapidFire Mass Spectrometry<br>LC-MS; powders re-ordered and independently synthesized and re-tested in concentration –response curves<br>All hits compounds reported were at least 85% pure by LC-MS.

Supplementary Table 1. h-cGAS high throughput small molecule screening data summary.

Molecule name	SMILES	Vendor	h-cGAS IC_{50} (µM)	m-cGAS IC_{50} (µM)
RU-0131894	CCN1CCC2=C(C1)SC(NC(=O)CC)=C2C1=NC2=CC=CC=C2S1	ChemDiv, Inc.	0.965	7.51
**RU-0179423 (J001)**	[O-][N+](=O)C1=C(NN=C1)C(=O)NC1=NC=C(Cl)C=C1	Vitas-M Laboratory, Ltd.	0.976	0.972
RU-0006086	FC(F)(F)C1=CC(=O)N2C3=C(NC2=C1C#N)C=CC=C3	Vitas-M Laboratory, Ltd.	1.05	17.9
RU-0168658	FC(F)(F)C1=NN2C(NC(CC3=CC=CC=C3)=CC2=O)=C1C1=CC=CC=C1	Vitas-M Laboratory, Ltd.	1.13	> 50.0
RU-0187170	NC(=O)N1C(O)=C(C(=O)C2=CC=CS2)C2=CC(Cl)=CC=C12	Tocris Bioscience	1.31	21.5
RU-0187094	CC1=C(C)C2=C(C=C1)C(=O)C1=CC=CC=C1N2CCCN	Tocris Bioscience	1.61	10.3
RU-0005637	FC(F)(F)C1=NC2=CC(Cl)=CC(Cl)=C2N1	Vitas-M Laboratory, Ltd.	2.39	> 50.0
RU-0166197	ClC1=CC=C2C(=C1)N=CC=C2N1C=CN=C1	Vitas-M Laboratory, Ltd.	2.64	10.8
**RU-0273458 (G001)**	COCC(=O)N1CCC2=C(C1)C1=CC(Cl)=CC(Cl)=C1N2	ENAMINE Ltd.	2.75	1.14
RU-0010201	OC(=O)CN1C(=S)S\C(=C\C2=C(O)C(Br)=CC(=C2)[N+]([O-])=O)C1=O	ChemBridge Corporation	3.02	5.77
RU-0209189	COC(=O)C1=NN2C(N1)=NS(=O)(=O)C1=CC=CC=C21	ENAMINE Ltd.	3.04	3.93
RU-0020238	OC(=O)C1=CC=CC=C1C1=CC=C(O1)\C=C1/SC(=S)NC1=O	Vitas-M Laboratory, Ltd.	3.78	9.65
RU-0183726	ClC1=C(Cl)C(NC(=O)C(C#N)C(=O)\C=C\C2=NC=CS2)=CC=C1	ENAMINE Ltd.	4.63	17.3
RU-0142869	CNC(=O)CSC1=NC(=O)N2N=C(C)C(=C2N1)C1=CC=CC=C1	Life Chemicals Inc.	4.78	> 50.0
RU-0169289	OC1=CC=C(\C=C2\C(=N)N3N=C(SC3=NC2=O)C2=C(Cl)C=CC=C2)C=C1	Vitas-M Laboratory, Ltd.	5.18	14.9
RU-0166710	O=C1N(NC(=C1\C=N/C1CC1)C1=CC=CC=C1)C1=NC2=CC=CC=C2S1	Specs	5.27	> 50.0
RU-0172034	CSC(=S)N\N=C(/C1=CC=CC=C1)C1=NC=CC=C1	Life Chemicals Inc.	7.02	> 50.0
RU-0003433	ClC1=C(SC2=CC=CC=C12)C1=NNC(=S)O1	Vitas-M Laboratory, Ltd.	7.16	16.1
RU-0208705	CC1=CC=C(C=C1)N1NC(=CC1=O)C(=O)NC1=CC=NC2=CC=NN12	ENAMINE Ltd.	7.82	> 50.0
RU-0002019	ClC1=C(Cl)C(Cl)=C2NC(=O)OC2=C1	ChemDiv, Inc.	8.1	11.6
RU-0001026	OC(=O)CC1=CC(I)=C(OC2=CC(I)=C(O)C(I)=C2)C(I)=C1	Vitas-M Laboratory, Ltd.	11.9	29.2
RU-0166755	CCC\N=C/C1=C(NN(C1=O)C1=NC2=CC=CC=C2S1)C1=CC=CC=C1	Specs	12.8	> 50.0
RU-0010562	OC1=C2N=C(NC3=CC=CC=N3)C=CC2=CC=C1	ChemDiv, Inc.	13.2	> 50.0
RU-0208404	OC1=CC=CN=C1NC(=O)C1=C2CCCCCN2C(=N1)C1=CC=CC=C1	ENAMINE Ltd.	13.7	> 50.0
RU-0093754	CC1=C2C(CCC3=C2C=CC=C3)=NC2=CC3=C(OCO3)C=C12	ENAMINE Ltd.	23.1	> 50.0
RU-0182153	OC1=CC=CC=C1C1=NNC2(CC3CC2C2CCCC32)S1	Vitas-M Laboratory, Ltd.	33.1	> 50.0
RU-0171678	COCC1=NN2C(NC(CC3=CC=CC=C3)=CC2=O)=C1C1=CC=CC=C1	Vitas-M Laboratory, Ltd.	> 50.0	> 50.0

Supplementary Table 2. Re-evaluation of freshly sourced non-intercalating compounds selected with IC_{50} < 10 µM in ATP-coupled luminescence assay and HPLC-MS measured purity of ≥ 85%. IC_{50}s were determined using RF-MS based assay.

	h-cGAS-G108	h-cGAS-G150	h-cGAS-cGAMP
**Data collection**			
Wavelength (Å)	0.9792	0.9792	0.9793
Space group	$P\,6_4$	$P\,6_4$	$P2_12_12$
Cell dimensions			
a, b, c (Å)	117.0, 117.0, 60.0	116.5, 116.5, 60.0	128.0, 50.4, 60.0
α, β, γ (°)	90, 90,120	90, 90,120	90, 90, 90
Resolution (Å)	29.43-2.45 (2.49-2.45)	29.35-2.40 (2.44-2.40)	29.98-2.60 (2.64-2.60)
R-pim	2.3 (38.4)	2.2 (28.5)	4.4 (16.6)
$I/\sigma I$	26.1 (1.5)	26.3 (2.4)	15.4 (4.5)
Completeness (%)	98.4 (89.6)	98.3 (93.2)	92.7 (97.9)
Redundancy	9.7 (4.8)	9.0 (4.4)	3.4 (3.1)
CC1/2	0.997 (0.582)	0.997 (0.737)	0.952 (0.949)
No. reflections	17109 (1635)	17953 (1719)	11586(1186)
Reflections used for R_{free}	1714 (164)	1796 (177)	1159 (119)
**Refinement**			
R_{work}/R_{free}	22.2/25.6	21.3/25.7	22.1/26.5
No. of non-hydrogen atoms			
Protein	2723	2703	2755
Ligand/Ion	25	27	46
Water	26	45	0
B-factors			
Protein	81.8	60.9	67.6
Ligand/Ion	102.0	72.5	74.7
Water	60.9	46.3	
R.m.s. deviations			
Bond lengths (Å)	0.003	0.004	0.004
Bond angles (°)	0.54	0.61	0.60
Ramachandran plots			
Favored (%)	96.0	96.9	96.6
Allowed (%)	4.0	2.8	3.4
Outliers (%)	0	0.3	0
Statistics for the highest-resolution shell are shown in parenthesis.			

Supplementary Table 3. X-ray statistics for h-cGAS-inhibitor or h-cGAS-cGAMP complexes.

Number of compounds	Vendor
53675	AMRI, (Albany, NY)
700	Analyticon Discovery, (Potsdam, Germany)
10150	Biofocus, (Charles River, Wilmington, MA)
4000	Cerep, (Poitiers, France)
64477	ChemBridge Corporation, (San Diego, CA)
23893	ChemDiv Inc., (San Diego, CA);
1335	ChemX-Infinity, (Romainville, France)
2094	Edelris, (Lyon, France)
81739	ENAMINE Ltd., (Monmouth Junction, NJ)
240	Greenpharma, (Órleans, France)
33026	Life Chemicals Inc, (Niagara-on-the-Lake, Canada)
1280	Sigma Aldrich, (St. Louis, Misouri)
694	NIH Clinical Collection, (NIH Small Molecule Repository)
905	Pharmakon-900, (MicroSource, Gaylordsville, CT)
1109	Prestwick, (Illkirch, France)
808	Selleckchem, (Houston, TX)
4051	Specs, (Zoetermeer, The Netherlands)
2000	Spectrum Chemicals, (New Brunswick, NJ)
480	Tocris Bioscience, (Bristol, UK)
761	Vitas-M Laboratory Ltd., (Kowloon, Hong Kong)

Supplementary Table 4. High Throughput and Spectroscopy Resource Center at The Rockefeller University library composition.

Gene	Species	Forward (5'→3')	Reverse (5'→3')
IFNB1	Human	GCTTCTCCACTACAGCTCTTTC	CAGTATTCAAGCCTCCCATTCA
TUBA1B	Human	ACCTTAACCGCCTTATTAGCCA	ACATTCAGGGCTCCATCAAATC
Ifnb1	Mouse	GATGACGGAGAAGATGCAGAAG	ACCCAGTGCTGGAGAAATTG
Tuba1a	Mouse	CCTACAATTCCATCCTCACCAC	CGCTCAATGTCGAGGTTTCT
CXCL10	Human	CCATTCTGATTTGCTGCCTTATC	TACTAATGCTGATGCAGGTACAG
ISG15	Human	CTCATCTTTGCCAGTACAGGAG	GGGACACCTGGAATTCGTT
TNF	Human	CCAGGGACCTCTCTCTAATCA	TCAGCTTGAGGGTTTGCTAC

Supplementary Table 5. List of primers used for quantitative RT-PCR.

Supplementary Methods

Synthesis of J014 (4-nitro-*N*-(4-phenylpyridin-2-yl)-1*H*-pyrazole-5-carboxamide)

Illustration of reaction scheme for the synthesis of **J014** is shown in Supplementary Figure 12a. To a solution of 4-nitro-1H-pyrazole-3-carboxylic acid (0.013 g, 80 µmol) in THF (1 ml) was added DMF (0.062 µl, 0.800 µmol) and oxalyl chloride (0.014 ml, 160 µmol). After being stirred at room temperature for 30 min, the mixture was evaporated by a stream of N_2 gas. 2-Amino-4-phenyl-pyridone (160 µmol) in DMA (0.5 ml) and DIEA (0.028 ml, 160 µmol) was added to the mixture. The mixture was stirred at room temperature for 3 h. The mixture was poured into water at room temperature and evaporated by a steady air stream at 60°C. The residue was purified by preparative HPLC (YMCTriartC18, eluted with MeCN/0.1%TFA to water/0.1%TFA). The desired fraction was air-dried at 60°C to obtain 4-nitro-*N*-(4-phenylpyridin-2-yl)-1*H*-pyrazole-5-carboxamide (27.23 mg).

LCMS: RT = 0.894 min, purity: 98.74%, *m/z* 309.1, 310.1 $[MS+H]^+$

Synthesis of G108 {1-(6,7-dichloro-9-(1*H*-pyrazol-4-yl)-1,3,4,5-tetrahydro-2*H*-pyrido[4,3-*b*]indol-2-yl)-2-hydroxyethan-1-one}.

An illustration of reaction scheme for the synthesis of G108 is shown in Supplementary Figure 12b. To a solution of compound **1** (20 g, 90.30 mmol, 1 eq) in dimethyl formamide (100 ml) was added compound **A** (7.56 g, 32.51 mmol, 0.36 eq) at 25°C. The mixture was stirred for 12 h at 50°C at which point TLC (petroleum ether: ethyl acetate=20:1) showed starting material was consumed and new spot was observed. The

mixture was poured into water (200 ml). The aqueous phase was extracted twice with ethyl acetate (100 ml each). The combined organic phase was washed 3 times with brine (100 ml each), dried over anhydrous sodium sulfate, filtered and concentrated in vacuum. The residue was triturated with petroleum ether (20 ml) at 0°C. The solid was collected by filtration to obtain compound **2** (20 g, 78.15 mmol, 86.54% yield) as a white solid.

^{1}H NMR: ($CDCl_3$, 400 MHz) δ 7.12 (d, *J* = 2.8 Hz, 1H), 7.00 (d, *J* = 2.8 Hz, 1H), 3.79 (s, 3H).

To a mixture of compound **2** (20 g, 78.15 mmol, 1 eq), compound **B** (18.40 g, 93.78 mmol, 1.2 eq), Xantphos (7.45 g, 15.63 mmol, 0.2 eq) and cesium carbonate (63.66 g, 195.37 mmol, 2.5 eq) in toluene (200 ml) was added palladium acetate (1.75 g, 7.81 mmol, 0.1 eq) at 25°C under nitrogen. The mixture was stirred for 10 h at 100°C under nitrogen. LCMS showed the starting material was consumed completely and the desired mass was detected. The mixture was cooled to 30°C and filtered, and the clear liquid was concentrated. The residue was purified by column chromatography (SiO_2, Petroleum ether = 1) to obtain compound **3** (20 g, 46.71 mmol, 59.77% yield, 86.70% purity) as a yellow solid.

LCMS: RT = 1.075 min, purity: 86.70%, *m/z* 371.0, 373.0 $[MS+H]^+$

^{1}H NMR: ($CDCl_3$, 400 MHz) δ 8.12 (br. s, 1H), 7.65 - 7.55 (m, 5H), 7.39 - 7.34 (m, 5H), 7.25 (d, *J* = 4.0 Hz, 1H), 6.53 (d, *J* = 4.0 Hz, 1H), 3.86 (s, 3H).

A solution of compound **3** (7 g, 18.85 mmol, 1 eq) in dioxane (60 ml) and hydrochloric acid (12M, 15 ml) was stirred for 1 h at 100°C. TLC (petroleum ether:ethyl acetate, 3:1) showed the starting material was consumed completely and several new spots were observed. The mixture was cooled to 0°C and adjusted to pH 8 by saturated sodium

bicarbonate solution. The aqueous phase was extracted 3 times with ethyl acetate (150 ml each). The combined organic phase was washed with brine (200 ml), dried with anhydrous sodium sulfate, filtered and concentrated in vacuum. The residue was purified by silica gel chromatography (petroleum ether:ethyl acetate=20:1~5:1) to give compound **4** (3 g, 14.32 mmol, 75.96% yield, 98.85% purity) as a yellow solid.

LCMS: RT = 0.509 min, purity: 98.845%, *m/z* 207.0, 208.9 $[MS+H]^+$

^{1}H NMR: (DMSO-$d_{6,}$ 400 MHz) δ 6.83 (br. s, 1H), 6.72 (d, *J* = 4.0 Hz, 1H), 6.41 (d, *J* = 3.2 Hz, 1H), 4.21 (br. s, 2H), 3.73 (s, 3H).

To a solution of compound **4** (6.5 g, 31.39 mmol, 1 *eq*) and ketone **C** (5.11 g, 37.67 mmol, 1.2 *eq*, HCl) in dioxane (70 ml) was added concentrated sulfuric acid (9.02 g, 91.92 mmol, 4.9 ml, 2.93 *eq*) at 0°C. The mixture was stirred for 12 h at 80°C. LCMS showed the starting material was consumed completely and desired compound mass was detected. The mixture was concentrated in vacuum. The residue was diluted with water (20 ml) and adjusted to pH 9 with sodium hydroxide solution (2 M) at 0°C. A precipitate appeared and was collected by filtration to give a crude product. The crude product was triturated with ethyl acetate (15 ml) to give compound **5** (6.5 g, 22.90 mmol, 72.94% yield, 95.51% purity) as a yellow solid.

LCMS: RT = 0.648 min, purity: 96.510%, *m/z* 271.0, 273.0 $[MS+H]^+$

^{1}H NMR: (DMSO-$d_{6,}$ 400 MHz) δ 11.64 (br.s, 1H), 6.73 (s, 1H), 4.30 (s, 2H), 3.86 (s, 3H), 3.56 (s, 2H), 2.96 (s, 2H),

To a solution of compound **5** (2 g, 7.05 mmol, 1 eq) in dichloromethane (50 ml) was added boron tribromide (5.30 g, 21.16 mmol, 2.04 ml, 3 eq) at 0°C. The mixture was stirred at 25°C for 12 h. LCMS showed the desired mass was detected. The mixture was

concentrated in reduced pressure. The residue was poured into saturated sodium bicarbonate solution (30 ml). The aqueous phase was extracted 3 times with ethyl acetate (30 ml each). The combined organic phase was washed with brine (10 ml), dried with anhydrous sodium sulfate, filtered and concentrated in vacuum to obtain compound **6** (1.8 g, 7.0 mmol, 99.24% yield) as a yellow solid.

^{1}H NMR: (DMSO-d_6, 400 MHz) δ 11.48 (br. s, 1H), 6.61 (s, 1H), 4.33 (s, 2H), 3.39 - 3.35 (m, 2H), 3.00 - 3.97 (m, 2H).

To a solution of compound **6** (1 g, 3.89 mmol, 1 *eq*) and triethylamine (1.18 g, 11.67 mmol, 1.62 ml, 3 *eq*) in tetrahydrofuran (20 ml) was added Boc_2O (679.07 mg, 3.11 mmol, 714.81 µl, 0.8 *eq*) at 25°C, then the mixture was stirred for 1 h at 25°C. LCMS showed the starting material was consumed completely and the desired mass was detected. The mixture was poured into ice-water (40 ml). The aqueous phase was extracted 3 times with ethyl acetate (20 ml each). The combined organic phase was washed twice with brine (20 ml each), dried with anhydrous sodium sulfate, filtered and concentrated in vacuum. The residue was purified by column chromatography (SiO_2, Petroleum ether: Ethyl acetate = 10: 1 to 3: 1) to give compound **7** (0.8 g, 2.13 mmol, 54.76% yield, 95.1% purity) as a yellow solid.

LCMS: RT = 0.864 min, purity: 95.065%, *m/z* 379.0 $[MS+Na]^+$

^{1}H NMR: ($CDCl_3$, 400 MHz) δ 8.16 (br. s, 1H), 7.90 (br. s, 1H), 6.48 (s, 1H), 4.83 (s, 2H), 3.81 (t, J = 6.0 Hz, 2H), 2.85 - 2.81 (m, 2H), 1.59 (s, 9H).

To a solution of compound **7** (700 mg, 1.96 mmol, 1 eq) in pyridine (5 ml) and dichloromethane (10 ml) was added Tf_2O (1.11 g, 3.92 mmol, 646.61 µl, 2 eq) at 0°C. The mixture was stirred for 1 h at 25°C. LCMS showed the reaction was completed. The

mixture was poured into ice-water (30 ml). The aqueous phase was extracted twice with ethyl acetate (20 ml each). The combined organic phase was washed with hydrochloric acid (1 M, 20 ml) and twice with brine (10 ml each), dried with anhydrous sodium sulfate, filtered and concentrated in vacuum to obtain compound **8** (900 mg, 1.84 mmol, 93.87% yield) as a yellow solid.

LCMS: RT = 1.001 min, purity: 89.383%, *m/z* 432.9, 434.9 $[MS-55]^+$

^{1}H NMR: ($CDCl_3$, 400 MHz) δ 8.43 (br. s, 1H), 7.16 (s, 1H), 4.76 (s, 2H), 3.83 (t, *J* = 5.6 Hz, 2H), 2.89 - 2.87 (m, 2H), 1.51 (s, 9H).

To a mixture of compound **8** (200 mg, 408.75 µmol, 1 eq), compound **D** (118.97 mg, 613.13 µmol, 1.5 eq) and potassium carbonate (169.48 mg, 1.23 mmol, 3 eq) in dioxane (10 ml) and water (3 ml) was added $Pd(dppf)Cl_2.CH_2Cl_2$ (33.38 mg, 40.88 µmol, 0.1 eq) at 25°C under nitrogen. The mixture was stirred for 10 h at 80°C. LCMS showed the starting material was consumed completely and the desired mass was detected. The mixture was filtered and the filter was poured into water (20 ml). The aqueous phase was extracted with 3 times with ethyl acetate (15 ml each). The combined organic phase was washed with brine (10 ml), dried with anhydrous sodium sulfate, filtered and concentrated in vacuum. The residue was purified by prep-TLC (SiO_2, Petroleum ether: Ethyl acetate = 2:1) to obtain compound **9** (150 mg, 362.76 µmol, 88.75% yield, 98.5% purity) as a yellow solid.

LCMS: RT = 0.850 min, purity: 98.497%, *m/z* 429.0, 431.0 $[MS+Na]^+$

^{1}H NMR: (CD_3OD, 400 MHz) δ 7.75 (s, 1H), 7.60 (s, 1H), 7.00 (s, 1H), 4.24 (s, 2H), 3.75 (s, 2H), 2.85 (t, *J* = 5.6 Hz, 2H), 1.20 (s, 9H).

A mixture of compound **9** (150 mg, 368.29 μmol, 1 eq) in trifluoroacetic acid (3 ml) and dichloromethane (10 ml) was stirred for 10 h at 25°C. LCMS showed the reaction was completed. The solution was concentrated in vacuum to obtain compound **10** (120 mg, crude, TFA) as a yellow solid. The crude product was used into the next step without further purification.

^{1}H NMR: (CD_3OD, 400 MHz) δ 11.48 (br.s, 1H), 8.01 (br. s, 1H), 7.99 (s, 1H), 7.81 (s, 2H), 7.10 (s, 1H), 4.06 (s, 2H), 3.58 (t, *J* = 6.4 Hz, 2H), 3.18 (t, *J* = 6.0 Hz, 2H)

To a solution of compound **10** (120 mg, 390.65 μmol, 1 eq) and triethylamine (118.59 mg, 1.17 mmol, 163.12 μl, 3 eq) in dichloromethane (2 ml) was added compound **E** (80.01 mg, 585.98 μmol, 63.00 μl, 1.5 eq) at 0°C. The mixture was stirred for 0.5 h at 25°C. LCMS showed the starting material was consumed completely and desired mass was detected. The residue was poured into saturated sodium bicarbonate solution (20 ml) and stirred for 15 min. The aqueous phase was extracted 3 times with ethyl acetate (15 ml each). The combined organic phase was washed with brine (10 ml), dried with anhydrous sodium sulfate, filtered and concentrated in vacuum to give compound **11** (150 mg, crude) as a yellow solid which was used into the next step without further purification.

Preparation of 1-(6,7-dichloro-9-(1*H*-pyrazol-4-yl)-1,3,4,5-tetrahydro-2*H*-pyrido[4,3-*b*]indol-2-yl)-2-hydroxyethan-1-one

To a solution of compound **11** (150 mg, 368.32 μmol, 1 eq) in tetrahydrofuran (3 ml) and water (1 ml) was added lithium hydroxide monohydrate (46.37 mg, 1.10 mmol, 3 eq) at 25°C. The mixture was stirred for 1 h at 25°C. LCMS showed the starting material was consumed completely and the desired mass was detected. The residue was poured into

ice-water (20 ml) and adjusted to pH 7 by hydrochloric acid (1 M). The aqueous phase was extracted 3 times with ethyl acetate (15 ml each). The combined organic phase was washed twice with brine (10 ml each), dried with anhydrous sodium sulfate, filtered and concentrated in vacuum. The residue was purified by prep-TLC (ethyl acetate:methanol, 20: 1) to obtain **G108** (43 mg, 111.69 μmol, 30.32% yield, 94.86% purity) as a yellow solid.

LCMS: RT = 1.831 min, purity: 94.864%, *m/z* 365.0 $[MS+H]^+$

^{1}H NMR: (CD_3OD, 400 MHz) δ 7.84 - 7.82 (m, 2H), 7.02 (d, *J* = 2.4 Hz, 1H), 4.56 (s, 1H), 4.46 (s, 1H), 4.31 (s, 1H), 4.19 (s, 1H), 4.06 (s, 1H), 3.95 (t, *J* = 5.6 Hz, 1H), 3.75 (t, *J* = 6.4 Hz, 1H), 2.95 - 2.89 m, 2H)

Synthesis of G140

Step 1:

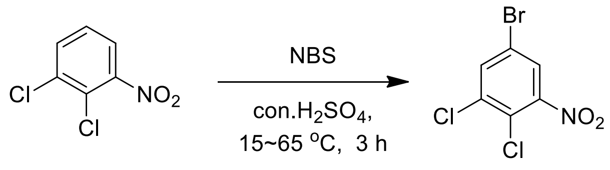

Step 1

5-bromo-1,2-dichloro-3-nitro-benzene

2 reactions were carried out in parallel

To a solution of 1,2-dichloro-3-nitro-benzene (50 g, 260.42 mmol, 1 *eq*) in H_2SO_4 (200 ml) was added NBS (55.62 g, 312.50 mmol, 1.2 *eq*) at 15 °C. The mixture was stirred at 65 °C for 3 h. The two reaction mixtures were combined. The reaction mixture was poured into ice water (1000 ml) and extracted with EtOAc(100 ml * 3). The combined organic layers were washed with brine (150 ml), dried over Na_2SO_4, filtered and concentrated under reduced pressure to give a crude product 5-bromo-1,2-dichloro-3-nitro-benzene (126.5 g, crude) as a yellow oil.

^{1}H NMR: ET16671-370-P1A (400MHz, METHANOL-d4)

δ 8.07 (d, *J* = 2.2 Hz, 1H), 7.81-7.61 (m, 1H)

Step 2:

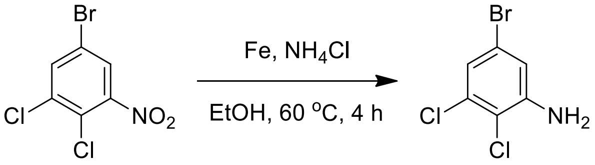

Step 2

5-bromo-2, 3-dichloro-aniline

To a solution of 5-bromo-1,2-dichloro-3-nitro-benzene (30 g, 110.74 mmol, 1 *eq*) in EtOH (400 ml) was added aq. NH_4Cl (0.33 M, 610.77 ml, 1.82 *eq*) and Fe (61.85 g, 1.11 mol, 10 *eq*). The mixture was stirred at 60 °C for 4 hr. The reaction mixture was filtered and concentrated under reduced pressure, extracted with EtOAc (50 ml * 3). The combined organic layers were washed with brine (80 ml), dried over Na_2SO_4, filtered and concentrated under reduced pressure to give a residue. The residue was purified by column chromatography (SiO_2, Petroleum ether) to give 5-bromo-2,3-dichloro-aniline (12 g, 49.81 mmol, 44.98% yield) as a yellow solid.

^{1}H NMR: ET16671-363-P1A (400MHz, CHLOROFORM-d)

δ 6.91 (d, *J* = 2.1 Hz, 1H), 6.74 (d, *J* = 2.2 Hz, 1H), 4.32-3.97 (m, 2H)

Step 3:

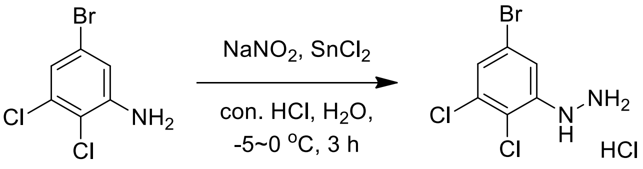

Step 3

(5-bromo-2, 3-dichloro-phenyl)hydrazine

Under a nitrogen atmosphere, a stirred solution of 5-bromo-2,3-dichloro-aniline (40 g, 166.04 mmol, 1 *eq*) in con. HCl (300 ml) was cooled to -5 °C, and a solution of $NaNO_2$ (17.18 g, 249.05 mmol, 1.5 *eq*) in H_2O (70 ml) was added drop-wise at a rate to keep the reaction mixture temperature under 0 °C. After the addition the reaction mixture was stirred for an additional 1 h at 0 to -5 °C. Then a solution of $SnCl_2.2H_2O$ (93.66 g,

415.09 mmol, 2.5 *eq*) in con. HCl (300 ml) was added drop-wise at a rate to keep the reaction mixture temperature under -5 to 0 °C. After that, the mixture was stirred at 0 °C for 2 h. The reaction mixture was filtered and the filter cake was washed with MTBE (50 ml), concentrated under reduced pressure to give a compound (5-bromo-2, 3-dichloro-phenyl)hydrazine (28 g, 109.41 mmol, 65.89 % yield) as a yellow solid.

^{1}H NMR: ET16671-367-P1A1 (400 MHz, DMSO-d6)
δ 7.31-7.21 (m, 2H), 7.00 (d, *J* = 2.32 Hz, 1H), 4.39 (br s, 2H)

Step 4:

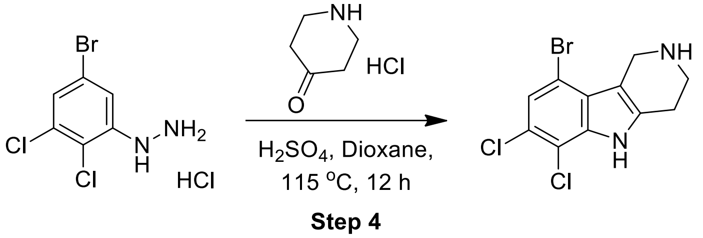

9-bromo-6, 7-dichloro-2, 3, 4, 5-tetrahydro-1H-pyrido[4,3-b]indole

To a solution of (5-bromo-2, 3-dichloro-phenyl)hydrazine (27.65 g, 94.57 mmol, 1 *eq*, HCl) and piperidin-4-one;hydrochloride (19.23 g, 141.85 mmol, 1.5 *eq*) in dioxane (500 ml) was added H_2SO_4 (192.09 g, 1.96 mol, 104.39 ml, 20.71 *eq*). The mixture was stirred at 115 °C for 12 hr. The reaction mixture was concentrated under reduced pressure. The residue was adjusted pH = 8 with NaOH (3 M), filtered and the filter cake was concentrated under reduced pressure to give a residue. The residue was washed with MTBE (50 ml), filtered and the filter cake was concentrated under reduced pressure to give a product 9-bromo-6,7-dichloro-2, 3,4,5-tetrahydro-1H-pyrido[4,3-b]indole (23.6 g, 62.69 mmol, 66.29% yield, 85% purity) as a gray solid.

^{1}H NMR: ET16671-378-P1A (400 MHz, DMSO-d6)
δ 7.36 (s, 1H), 4.15 (s, 2H), 3.01 (t, *J* = 5.48 Hz, 2H), 2.73-2.66 (m, 2H)

Step 5:

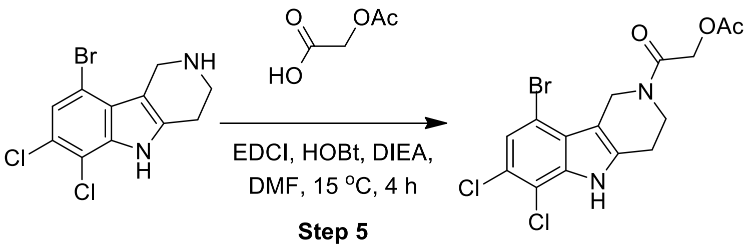

[2-(9-bromo-6, 7-dichloro-1,3,4,5-tetrahydropyrido[4,3-b]indol-2-yl)-2-oxo-ethyl] acetate

To a solution of 9-bromo-6,7-dichloro-2,3,4,5-tetrahydro-1H-pyrido[4,3-b]indole (1 g, 3.12 mmol, 1 *eq*) and 2-acetoxyacetic acid (442.81 mg, 3.75 mmol, 1.2 *eq*) in DMF (10 ml) was added EDCI (898.56 mg, 4.69 mmol, 1.5 *eq*), HOBt (633.35 mg, 4.69 mmol, 1.5 *eq*) and DIPEA (1.21 g, 9.37 mmol, 1.63 ml, 3 *eq*). The mixture was stirred at 15 °C for 4 h. The reaction mixture was diluted with H_2O (20 ml) and extracted with EtOAc (20 ml * 3). The combined organic layers were washed with brine (30 ml), dried over Na_2SO_4, filtered and concentrated under reduced pressure to give a residue. The residue was purified by column chromatography (SiO_2, Petroleum ether/Ethyl acetate = 3/1 to 1/4) to give [2-(9-bromo-6,7- dichloro-1,3,4,5-tetrahydropyrido[4,3-b]indol-2-yl)-2-oxo-ethyl] acetate (0.65 g, 1.41 mmol, 45.06% yield, 91% purity) as a yellow solid.

^{1}H NMR: ET16671-405-P1A1 (400MHz, DMSO-d6)
δ 11.95-11.81 (m, 1H), 7.41 (s, 1H), 4.92-4.81 (m, 4H), 3.84-3.65 (m, 2H), 2.93-2.74 (m, 2H), 2.07 (s, 3H)

Step 6:

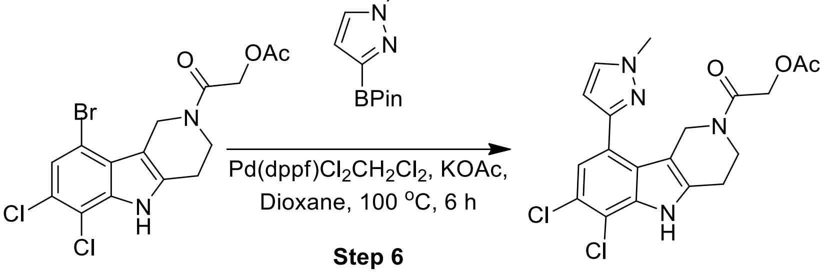

[2-[6,7-dichloro-9-(1-methylpyrazol-3-yl)-1,3,4,5-tetrahydropyrido[4,3-b]indol-2-yl]-2-oxo-ethyl] acetate

To a solution of [2-(9-bromo-6,7-dichloro-1,3,4,5-tetrahydropyrido[4,3-b]indol-2-yl)-2-oxo-et hyl] acetate (0.25 g, 595.12 µmol, 1 *eq*) and 1-methyl-3-(4,4,5,5-tetramethyl-1,3,2-dioxaborol a n-2-yl)pyrazole (247.65 mg, 1.19 mmol, 2 *eq*) in dioxane (20 ml) was added KOAc (175.21 mg, 1.79 mmol, 3 *eq*) and Pd(dppf)Cl_2.CH_2Cl_2 (97.20 mg, 119.02 µmol, 0.2 *eq*). The mixture was stirred at 100 °C for 6 h. The reaction mixture was concentrated under reduced pressure to give a residue. The residue was purified by column chromatography (SiO2, DCM/MeOH = 1/0 to 24:1) to give [2-[6,7-dichloro-9-(1-methylpyrazol-3-yl)-1,3,4,5-tetrahy dropyrido[4,3-b]indol-2-yl]-2-oxo-ethyl] acetate (0.5 g, crude) as a brown oil.

^{1}H NMR: ET16671-383-P1B (400 MHz, CHLOROFORM-d)
δ 8.26-8.40 (m, 1H) 7.39-7.30 (m, 2H) 7.20-7.16 (m, 1H) 6.41-6.31 (m, 1H) 4.67 (br s, 1H) 4.57-4.52 (m, 1H) 3.94 (s, 3H) 3.92-3.85 (m, 4H) 2.92-2.78 (m, 3H) 2.15-2.04 (m, 5H)

Step 7:

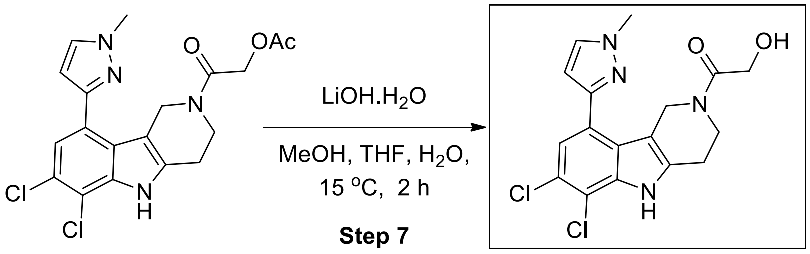

T015T-010-1

1-[6,7-dichloro-9-(1-methylpyrazol-3-yl)-1,3,4,5-tetrahydropyrido[4,3-b]indol-2-yl]-2-hydroxy-ethanone

To a solution of [2-[6,7-dichloro-9-(1-methylpyrazol-3-yl)-1,3,4,5-tetrahydropyrido[4,3-b]ind ol-2-yl]-2-oxo-ethyl] acetate (0.5 g, 1.19 mmol, 1 *eq*) in MeOH (10 ml), THF (10 ml) and H_2O (20 ml) was added LiOH.H_2O (149.40 mg, 3.56 mmol, 3 *eq*). The mixture was stirred at 15 °C for 2 h. The reaction mixture was diluted with H_2O (10 ml) and extracted with EtOAc (10 ml * 3). The combined organic layers were washed with brine (50 ml), dried over Na_2SO_4, filtered and concentrated under reduced pressure to give a residue. The residue was purified by prep-HPLC (neutral condition) to give 1-[6,7-

dichloro-9-(1-methylpyr azol-3-yl)-1,3,4,5-tetrahydropyrido[4,3-b]indol-2-yl]-2-hydroxy-ethanone (0.07 g, 184.58 µmol, 15.55% yield, 100.00% purity) as a white solid.

LCMS: ET16671-396-P1A ($M+H^+$): 379.0 @ 2.604 min (5-95% ACN in H_2O, 4.5 min)

^{1}H NMR: ET16671-396-P1A2 (400MHz, DMSO-d6)
δ 11.33 (br s, 1H), 7.73 (d, *J* = 2.2 Hz, 1H), 7.22 (s, 1H), 6.52 (br s, 1H), 4.57 (s, 2H), 4.32 - 4.26 (m, 1H), 4.13 (br s, 2H), 3.94 (s, 3H), 3.76 (br s, 2H), 2.88 (br s, 2H)
