# Supplementary material for: Development of human cGAS-specific small-molecule inhibitors for repression of dsDNA-triggered interferon expression
Source: Nat Commun. 2019 May 21;10:2261. doi: 10.1038/s41467-019-08620-4 (PMC6529454; doi:10.1038/s41467-019-08620-4)
Supplement: Supplementary file 3 — Description of Additional Supplementary Files [file 41467_2019_8620_MOESM3_ESM.pdf]

Description of Additional Supplementary Files

File Name: Supplementary Data 1
Description: DNA intercalation assay results for 100 non-PAIN screening hit compounds showing IC50 ≤ 10 μM in chemiluminescence (LUM) assay. Powder form for compounds in blue text re-ordered and re-tested using RF-MS assay as shown in Supplementary Table 3.
